# Evaluation of the Value of Histological Examination for the Prediction of Genetic Thoracic Proximal Aortopathies

**DOI:** 10.3390/jcm13071838

**Published:** 2024-03-22

**Authors:** Adrian Mahlmann, Roman N. Rodionov, Christian-Alexander Behrendt, Jennifer Lynne Leip, Helmut Karl Lackner, Mohamed Eraqi, Nesma Elzanaty, Tamer Ghazy

**Affiliations:** 1 Department of Internal Medicine III, University Hospital Carl Gustav Carus at Technische Universität, 01307 Dresden, Germany; mahlmanna@kkh-hagen.de (A.M.); roman.rodionov@ukdd.de (R.N.R.); 2 Centre for Vascular Medicine, Clinic of Angiology, St.-Josefs-Hospital, Katholische Krankenhaus Hagen gem. GmbH, 58097 Hagen, Germany; 3 University Center for Vascular Medicine, University Hospital Carl Gustav Carus, Technische Universität Dresden, 01307 Dresden, Germany; 4 Department of Vascular and Endovascular Surgery, Asklepios Clinic Wandsbek, Asklepios Medical School, 20099 Hamburg, Germany; behrendt@hamburg.de; 5 Brandenburg Medical School Theodor Fontane, 16816 Neuruppin, Germany; 6 Northeastern University, Boston, MA 02115, USA; leip.j@husky.neu.edu; 7 Division of Physiology, Otto Loewi Research Center for Vascular Biology, Immunology and Inflammation, Medical University of Graz, 8010 Graz, Austria; helmut.lackner@medunigraz.at; 8 Department of Cardiac Surgery, Klinikum Bayreuth GmbH, 95445 Bayreuth, Germany; mohamed.eraqi@klinikum-bayreuth.de; 9 Department of Medical Physiology, Tanta Faculty of Medicine, Tanta University, Tanta 31527, Egypt; nesma.elzanaty@med.tanta.edu.eg; 10 Department of Cardiac Surgery, Marburg University Hospital, Philipps University of Marburg, 35037 Marburg, Germany

**Keywords:** thoracic proximal aneurysm and dissection, connective tissue disease, histological and genetic findings

## Abstract

**Background**: Heritable connective tissue disorders are often accompanied by an increased risk for thoracic aortic aneurysm and dissection (TAAD). Profound knowledge of the underlying pathology may have an impact on individual treatment, systematic follow-up, and early detection by the screening of offspring. The aim of this study, based in a single high-volume tertiary center, was an analysis of the diagnostic validity of histopathologic findings in patients with TAAD due to these findings’ accuracy in diagnosing heritable connective tissue disorders. **Methods**: Therefore, genetic testing by next-generation sequencing (NGS) was performed to evaluate the correlations. In total, 65 patients with TAAD undergoing surgical treatment before the age of 60 years or with age up to 80 years if they had offspring at the time of the procedure were included in the analysis. **Results**: In our cohort, no certain correlation of histological findings to the results of genetic diagnostics in patients with clinically relevant aortic pathology could be shown. Patients with histopathologic findings for heritable connective tissue disorder and a positive gene variant were 11.6 years younger than patients without mutation and without histological evidence for connective tissue disorder. **Conclusions**: Genetic clarification is useful to define the specific genotype of the disease of the aortic wall in the case of non-specific histological characteristics.

## 1. Introduction

Thoracic aortic aneurysm and dissection (TAAD) and their association with high morbidity and mortality, involving not-seldom fatal consequences for affected patients, continue to pose significant challenges in the clinical setting today regarding prevention, diagnosis, and therapy. The incidence of TAAD varies depending on the publication and geographic location, ranging from 2.6 to 7.2 per 100,000 individuals. The true incidence rate might be underestimated because of sudden death events related to aortic diseases, which are not confirmed diagnostically by autopsy [1,2,3,4,5].

The etiology of TAAD is multifactorial. Common causes include cardiovascular risk factors, inflammatory diseases, mechanical forces such as deceleration trauma, and iatrogenic complications; amphetamine or cocaine abuse can also be a causative factor. It is also believed that approximately 20% of TAAD cases arise due to a genetic connective tissue disorder (CTD), which leads to instability in the walls of large arterial vessels [1,3].

TAAD caused by CTD represents a heterogeneous group of inherited or sporadic genetic variants that affect the stability of various tissues and can lead to clinically significant complications. Starting from a clinical suspicion, histological features can provide clues to the presence of a connective tissue disorder with aortic involvement, and further confirmation can be achieved through human genetic testing.

In recent years, the technology of high-throughput sequencing, also known as next-generation sequencing (NGS), has become available for genetic variant diagnostics in the human genome. NGS is comparable to the standard Sanger sequencing (with sensitivity ranging from 95.8% to 98.5% and specificity ranging from 98.1% to 98.8%) while offering technical superiority [6,7,8].

It is assumed that there might be diagnostic uncertainty regarding the histological findings for the estimation of the presence of a connective tissue disease in TAAD. Therefore, further genetic clarification is useful to define the specific genotype of the disease of the aortic wall in the case of non-specific histological characteristics. 

However, few data exist in the literature studying the correlation of genetic variants with histological findings in the diseased aorta. With the introduction of next-generation sequencing (NGS), we hypothesized that more genetic variants can be detected through NGS than through histological findings, and patients with genetic variants could pass through unnoticed if the diagnosis, or at least the indication for genetic sequencing, depends on positive histological findings. Therefore, this study aims to compare the correlation of histological findings to the results of human genetic diagnostics in patients with a clinically relevant aortic pathology.

## 2. Materials and Methods

### 2.1. Ethical Statement

This study was reviewed and approved by the ethical committee board of the Technische Universität Dresden (decision number EK317082014). The indication for human genetic analysis was made by the Department of Angiology at the University Centre for Vascular Medicine. It was also confirmed by the cooperating practice for human genetics. 

### 2.2. Patient Recruitment

All patients who underwent an open cardiosurgical procedure for TAAD in a single high-volume tertiary center were screened for inclusion in the study for genetic and histological diagnostics. The screening was based on the patients’ digital records at the Department of Cardiac Surgery, as well as the digital follow-up data records with the tertiary hospital’s aortic board. The inclusion criteria included undergoing the procedure before the age of 60, or up to 80 years but with offspring at the time of the procedure, with individual consequences for follow-up, reintervention regarding aortic disease, or screening for offspring, excluding a traumatic, inflammatory, or iatrogenic etiology; submitted histological specimens at the time of the procedure; surviving the procedure and the follow-up period; and adherence to the scheduled follow-up visits. 

Patients who met the inclusion criteria were then contacted for consent for inclusion in the study per the Genetic Diagnosis Act. Patients who failed to consent and patients with incomplete records, or incomplete histological or genetic analysis findings, were excluded from the study.

Data were collected for a total of 1064 patients who underwent open-heart surgery from August 2011 to August 2021. A total of 158 surviving patients, who were referred to the University Vascular Center Dresden for follow-up examinations, were contacted. Of these, 74 patients consented to participate in the study, and genetic findings from 65 of them were used for evaluation after obtaining renewed consent under the Genetic Diagnosis Act.

### 2.3. Histological Evaluation of Aortic Wall Specimens

Histopathological examinations of the intraoperatively obtained and submitted aortic resection specimens were performed for all included patients. The histological findings were analyzed for features indicative of the presence of a genetic connective tissue disorder. 

The specimens were examined by the pathologist on a macroscopic and microscopic level. The macroscopic examination was for the overall size and any abnormalities suggestive of atherosclerosis or dissection. The microscopic examination involved the use of various histological staining techniques; the staining techniques primarily included Elastica–van Gieson, Alcian blue, and Prussian blue staining. Elastica–van Gieson staining is a combination of Weigert’s elastic stain and van Gieson’s stain. This staining is the classical connective tissue stain, especially for elastic fibers. In this method, elastic fibers are stained blue-violet, muscle fibers yellow, and collagen fibers red in a two-step process. This staining allows for an assessment of the tissue’s stability and flexibility. Alcian blue staining is used to detect acidic proteoglycans and polysaccharides in the extracellular matrix. It uses a dye that stains glycosaminoglycan side chains cyan. Additionally, a Prussian blue reaction was applied to detect iron deposits, especially hemosiderin. The phagocytosis of hemoglobin by macrophages takes approximately 3 days, allowing the formed hemosiderin to provide insights into older sources of bleeding. The histological findings were analyzed for features indicative of the presence of a genetic connective tissue disorder as listed in Table 1, according to Halushka et al. [9].

### 2.4. Genetic Testing

A human genetic examination using next-generation sequencing was conducted as a part of routine diagnostics through our collaborating partner, the Practice for Human Genetics. To sequence the DNA, it was extracted from a sample of EDTA whole blood and then analyzed using NGS. After DNA fragmentation, the enrichment of probes was initially performed using captures through complementary binding to the target gene regions under examination. This was followed by clonal amplification, and the results were ultimately analyzed using the MiSeq Desktop Sequencer© (Illumina Inc., San Diego, CA, USA). 

Biometric data analysis was conducted for the genes listed in Table 2, modified from Faggion Vinholo et al. [10], including the copy number variation (CNV), which enables the detection of larger deletions or duplications.

For the classification of the human genetic findings, an adapted classification according to Plon et al. [11] is presented in Table 3.

### 2.5. Scoring System for the Clinical Assessment of Cardiovascular and Histopathological Findings

To assess the clinical probability regarding the overall cardiovascular risk and the presence of a structural connective tissue disorder, a point system was introduced. This system considers the presence of a diagnosis but not its severity.

For all patients, risk factors related to the occurrence of cardiovascular diseases were collected to assess the overall risk. The established score ranges from 0 to 4 and includes the following:

→ Arterial hypertension = 1 point;
→ Hyperlipoproteinemia = 1 point;
→ Current or past nicotine abuse = 1 point;
→ Diabetes mellitus = 1 point.

Additionally, cardiovascular diseases diagnosed as comorbidities in patients were recorded. These include coronary artery disease (CAD), peripheral arterial occlusive disease (PAOD), carotid stenosis/stroke, chronic obstructive pulmonary disease (COPD), and chronic renal insufficiency.

To evaluate the histopathological findings, a point system was also introduced for the respective pathologies to assess the severity of the detected changes individually and in comparison with other patients:

→ Presence of media degeneration = 1 point;
→ Changes in the extracellular matrix = 1 point;
→ Changes in elastic fibers = 1 point;
→ Changes in smooth muscle cells = 1 point;
→ Changes in collagen tissue = 1 point;
→ Summarized assessment by the pathologist regarding whether the findings may correspond to a genetic connective tissue disorder = 1 point.

Cumulatively, this results in a point value of 0–6, assuming a higher likelihood of a structural connective tissue disorder with an increasing score. Due to the heterogeneity of histopathological findings by different examiners, the severity of changes in individual categorical features could not be analyzed and considered in this study. The extent of atherosclerotic changes, including their severity, was separately recorded.

### 2.6. Statistical Analysis

A statistical analysis was conducted using the OriginPro software from OriginLab (OriginPro, Version 2022. OriginLab Corporation, Northampton, MA, USA). Statistical significance was assumed at a *p*-value of <0.05.

For descriptive analyses, ordinal data are presented as absolute values, and metric data are presented as means with standard deviations. The analysis of continuous variables was performed using an unpaired *t*-test, and for categorical variables, Pearson’s Chi-square test was used. An analysis of variance (ANOVA) was applied to examine the relationship between metric and ordinal data.

## 3. Results

### 3.1. Baseline Characteristics of Patients

The patients included in the analysis (*n* = 65) were, on average, 48.4 ± 10.6 years old at the time of surgical treatment for a thoracic aortic dissection (TAD in 55.4%, of which 77.8% had type A aortic dissection and 22.2% had type non-A and non-B aortic dissection) or aneurysm (TAA in 44.6%) and were predominantly of male gender (76.9% men compared to 23.1% women). All dissections and aneurysms involved the proximal aorta (the root, ascending segment, or arch of the aorta). Patients with a bicuspid aortic valve (10.8%) had aortic regurgitation more often than did those with a tricuspid valve (57.1 vs. 18.5%). The cardiovascular risk factors treated with medication and atherosclerotic comorbidities were recorded. Eighty percent of the patients had arterial hypertension, and most commonly, 12.3% of the patients had pre-existing coronary heart disease (Table 4).

**Figure 1:**
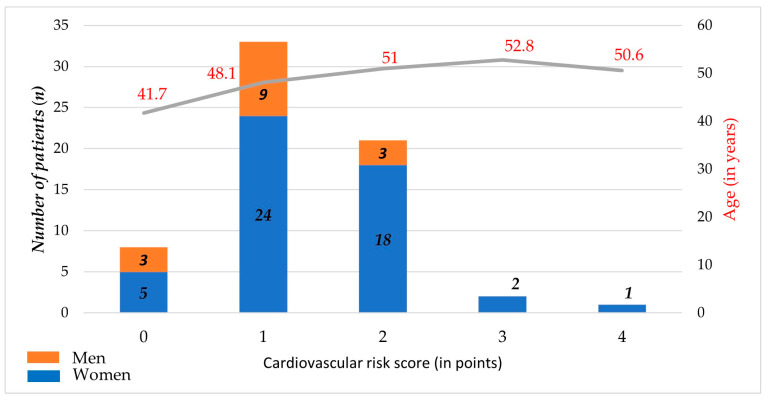
Cardiovascular risk score by age and gender.

The overall proatherogenic risk tended to increase with increasing age (*p* = 0.030) regardless of gender (*p* = 0.545, n.s.) (Figure 1).

### 3.2. Histologic Findings

In 29 patients (61.7% of the study cohort), a connective tissue disease was suspected by the pathologist based on all histological signs. Media degeneration and changes in elastic fibers were diagnosed most frequently (80.9% each), with fragmentation and/or loss of elastic fibers leading the way (63.8%). In 66.0%, there was a change in the extracellular matrix due to the incorporation of acidic mucopolysaccharides, being of severe grade in most cases (31.9%). In addition, 51.1% of patients had atherosclerotic changes, mostly of mild grade (36.2%). Changes in the vascular smooth muscle (6.4%) and collagen structure (2.1%) were rarely diagnosed without further differentiation (Table 5).

### 3.3. Genetic Results

The included patients were examined using next-generation sequencing (NGS) diagnostics for known mutations that, according to the state of scientific knowledge at the time of analysis, are known to be causally related to aortic connective tissue disease and thoracic aortic pathologies such as TAD and/or TAA. Such a mutation was detected in 46.2%, regardless of gender. Three carriers had mutations in two different gene locations. Patients with a detected mutation were, on average, 4.5 years younger than those without mutation (Table 6).

In 5 cases (of the 30 patients with a detected gene mutation, 16.7%), a classic mutation in genes related to aortic connective tissue disease and thoracic aortic pathology was detected. In the majority (27 cases), genetic variants were diagnosed (Table 7).

### 3.4. Correlation between Histological and Human Genetic Findings

**Figure 2:**
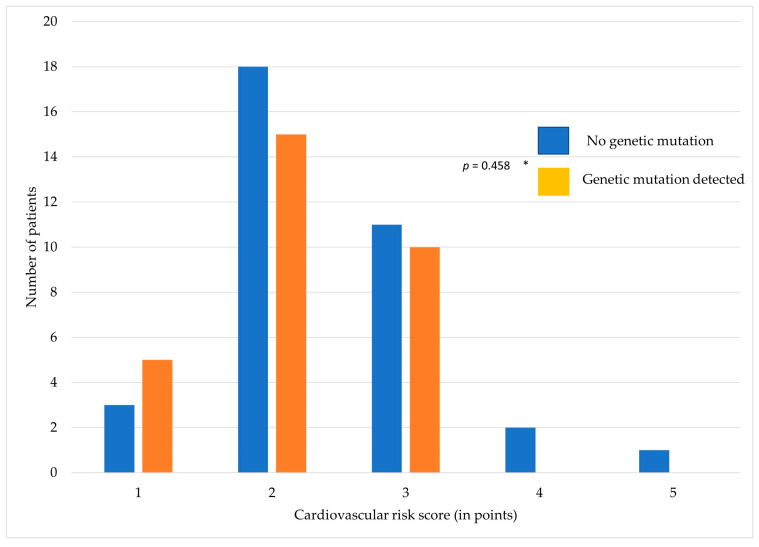
Relationship between cardiovascular risk score and detection of gene mutation.

Patients with a detected mutation in genes associated with aortic connective tissue disorders showed a lower cardiovascular risk score compared to patients without any such genetic mutation (*p* = 0.458) (Figure 2).

**Figure 3:**
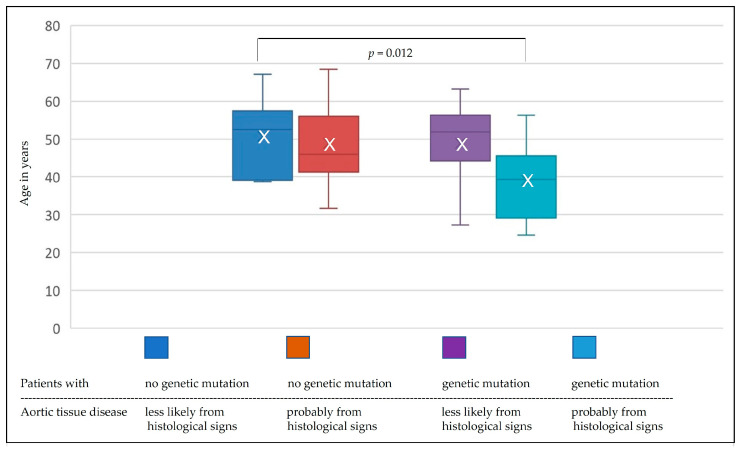
Differentiated analysis of age depending on the results of histological and genetic analyses.

Patients with histological criteria probably related to aortic connective tissue disease and thoracic aortic pathology and subsequently confirmed evidence of a gene mutation were, on average, 11.6 years younger (39.1 ± 9.8 vs. 50.7 ± 10.4 years; *p* = 0.012) than the patients without genetic mutation and without histological signs indicative of aortic connective tissue disease (Figure 3).

Histological signs indicative of aortic tissue disease were found to not be reliably predictive of the results from NGS diagnostics (*p* = 0.074, n.s.).

## 4. Discussion

The management of thoracic aortic aneurysm and dissection (TAAD) is still challenging, as epidemiological data on thoracic aortic aneurysm and dissection (TAAD) are partly underestimated relative to the true incidence because of sudden death events that are related to aortic diseases but not confirmed diagnostically by autopsy. Also, the respective risk of rupture and corresponding acute management in cases of acute aortic syndrome are quite different in the case of involvement of the ascending aorta. Recently, the YALE research group described an increased relative risk of adverse events in unoperated patients when the aneurysm surpasses 5 cm, with overall slow aortic growth [12]. The aortic maximal diameter may also show a correlation with histopathological findings and possibly with genetic mutations, and it clearly affects the risk of acute aortic events if it is >5 cm. The underlying pathology of the aortic wall is heterogeneous, with up to 20% of cases involving suspected genetic aortopathy [1]. In addition to the patient’s phenotype and familial predisposition to aortic diseases, pathophysiological findings and indications of a possible connective tissue disease can be obtained through histological examination of the aortic specimen preserved during cardiac surgery. In recent years, the Society for Cardiovascular Pathology (SCVP) and the Association for European Cardiovascular Pathology (AECVP) have declared a structured nomenclature and diagnostic criteria for the uniform histological assessment of non-inflammatory, degenerative aortic preparations to ensure comparability [9]. In addition, genetic diagnostics by next-generation sequencing (NGS) can be performed on patients to clarify the genesis of the disease of the aortic wall in TAAD. Up to now, there have been no valid considerations of reliable predictions from histological criteria indicative of aortic tissue diseases to the results of genetic testing of patients by NGS in known gene locations associated with connective tissue diseases. This study aimed to demonstrate real-world data of unselected patients undergoing cardiac surgery due to TAAD, to show the diagnostic uncertainty of histology alone and the importance of a required genetic examination for a better understanding of aortic diseases.

According to the guidelines for the diagnosis and treatment of aortic diseases, patients with TAAD have a mean age of 63 years, although women tend to develop the disease at an older age. In old age, atherosclerosis and its cardiovascular risk factors are the main cause of the pathogenesis of diseases of the aortic wall, whereas when it first manifests at a younger age (usually <60 years), a connective tissue disease is suspected [4,13,14]. In our study, the mean age of the patients, who were predominantly of male gender, was 48.4 ± 10.6 years, so genetic aortopathy was statistically probable according to the previous literature. All aortic dissections and aneurysms were located in the proximal thoracic aortic segments like the root, ascending aorta, or arch. Root dilatation and supracoronary disease of the ascending aorta are more commonly correlated to genetically driven pathology than to the distal thoracic or thoracoabdominal aorta. Also, it is known that the morphology of aneurysms of the ascending aorta differs markedly from that of aneurysms of the descending aorta (which are more related to atherosclerosis) [15]. Sixty-two percent of the patients showed histopathological signs of aortic connective tissue disease, mostly media degeneration and changes in the elastic fibers, in 80.9% each. These histological findings also correspond to the results of other works [16]. In publications on histological examinations of TAAD, patients were assigned to different syndromes, although it usually remained unclear whether this diagnosis was made after human genetic examination or solely based on clinical and phenotypological aspects [17,18]. Among others, Waters et al. [19] evaluated the structured histological nomenclature in TAAD and concluded that this can contribute to the differentiation of syndromic and non-syndromic aortopathy [20]. Histological features such as media degeneration are nonspecific and can occur in atherosclerosis as well as in various genetic aortopathies [9,20]. Therefore, genetic examinations, such as next-generation sequencing, are mandatory for further clarification. The knowledge about gene loci with a possible association with thoracic aortopathy in connective tissue disease is constantly advancing [21]. In our present study, we were able to detect such mutations causally related to aortic connective tissue disease and thoracic aortic pathology (TAAD) in 46.2% of patients, with genetic variants being more common than classic mutations. The relevance of variants of unknown significance (VUS) is a hot topic in research to clarify their impact on aortic diseases [22,23]. Even though histological signs indicative of aortic tissue disease were not found to be reliably predictive of the results from NGS diagnostics, the combined effect of both findings was shown. Patients with histological findings probably related to aortic connective tissue disease and thoracic aortic pathology and subsequently confirmed evidence of a gene mutation were, on average, 11.6 years younger than the patients without genetic mutation and without histological signs indicative of aortic connective tissue disease. Further studies in a larger cohort are needed to gain a better pathophysiological understanding of genetic variants with an assessment of their clinical relevance. As part of the clinical routine, patients with TAAD should be characterized phenotypically and by histological and genetic examinations, especially if a syndromic connective tissue disease is clinically suspected, in cases of sporadic TAAD at an age of <60 years at first manifestation or with a positive family history of familial TAAD (FTAAD), with and without mutation detection. But even at an age of >60 years, a human genetic evaluation can be useful if it has consequences for the patient themselves, like for planning a structured follow-up, for the differential therapeutic selection of an adequate treatment method, or for the screening of offspring.

## 5. Limitations

The results of our study are limited by the small number of patients. The underlying pathomechanism differs in aortic aneurysm and dissection. Further investigations should deeply focus on the differences between these two categories. There were also differences in the evaluation of the histological findings by several pathologists. A defined standard is essential, especially in regard to grading histological signs.

## Figures and Tables

**Table 1:** Diagnosis criteria and assessment for the evaluation of a present connective tissue disorder.

Diagnostic Criteria	Microscopic Histological Assessment
Overall media degeneration	Grading: none, mild, moderate, severe
Changes in the extracellular matrix	Mucoid extracellular matrix accumulation (MEMA)
Changes in elastic fibers	Elastic fiber fragmentation and/or loss (EFF) Elastic fiber thinning Elastic fiber disorganization (EFD)
Changes in smooth muscle cells	Smooth muscle cell nucleus loss (SMCL) Smooth muscle cell disorganization Laminar medial collapse (LMC)
Changes in collagen tissue	Medial fibrosis
Presence of atherosclerosis	Grading: mild, moderate, severe
Summary assessment by the pathologist as to whether the findings may correspond to a genetic connective tissue disorder	Concordant, nonconcordant

**Table 2:** Examined gene locations associated with syndromic or non-syndromic thoracic aortic diseases with aneurysm and/or dissection.

Gene name	Protein	Associated (Aortic) Disease/Syndrome
ACTA2 (NM_001613.2)	Smooth muscle α-actin	TAAD (thoracic aortic aneurysm and dissection)
AEBP1 (NM_001129.4)	AE binding protein 1	Abdominal aortic aneurysm (AAA) Ehlers–Danlos syndrome (EDS)
BGN (NM_01711.5)	Biglycan	TAAD Meester–Loeys syndrome
COL1A1 (NM_000088.3)	Collagen 1 α1 chain	TAAD EDS
COL3A1 (NM_000090.3)	Collagen 3 α1 chain	TAAD EDS, vascular Type (IV)
COL4A5 (NM_000495.3)	Collagen 4 α5 chain	TAAD Defect collagen Type IV, Alport syndrome
COL5A1 (NM_000093.4)	Collagen 5 α1 chain	TAA (thoracic aortic aneurysm) EDS, classical Type I
COL5A2 (NM_000393.4)	Collagen 5 α2 chain	TAA EDS, classical Type II
EFEMP2 (FBLN4) (NM_016938.4)	Pin-4	TAA, other arterial aneurysms Skin laxation, (autosomal recessive) Type Ib
ELN (NM_000501.3, NM_001278939.1)	Elastin	TAAD Loose skin (autosomal dominant)
FBLN5 (NM_006329.3)	Pin 5	TAAD Cutis laxa, macular degeneration
FBN1 (NM_000138.4)	Fibrillin-1	TAAD, AAA, and other arterial aneurysms Marfan syndrome
FBN2 (NM_001999.3)	Fibrillin-2	TAAD
FLNA (NM_001110556.2)	Filamin A	TAAD
FOXE3 (NM_012186.2)	Forkhead box	TAAD
GATA5 (NM_080473.4)	GATA binding protein	TAA
LOX (NM_002317.6)	Lysyl oxidase	AAA/dissection
MAT2A (NM_005911.5)	Methionine adenosyl -transferase II α	TAA
MFAP5 (NM_003480.3)	Microfibril-associated glycoprotein 2	TAAD
MYH11 (NM_002474.2)	Smooth muscle myosin heavy chain	TAAD
MYLK (NM_053025.3)	Myosin light chain kinase	TAAD
NOTCH1 (NM_017617.4)	Notch receptor 1	TAAD
NOTCH3	Notch receptor 3	TAAD
PLOD1 (NM_000302.3)	Procollagen-lysine,2-oxoglutarate 5-dioxygenase 1	TAAD EDS
PRKG1 (NM_006258.3)	Type I cGMP-dependent protein kinase	TAAD, AAA
SKI (NM_003036.3)	Sloan Kettering proto-oncoprotein	TAA Shprintzen–Goldberg syndrome
SLC2A10 (NM_030777.3)	Glucose transporter 10	TAA, other arterial aneurysms Arterial torsion syndrome
SMAD3 (NM_005902.3)	SMAD3	TAAD, AAA, other arterial aneurysms Loeys–Dietz syndrome Type III
SMAD4 (NM_005359.5)	SMAD4	TAAD
SMAD6 (NM_005585.4)	SMAD6	TAA
TAB2 (NM_015093.5)	TGF-beta activated kinase 1 (MAP3K7) binding protein 2	TAAD
TGFB2 (NM_003238.4)	TGF-β2	TAAD Loeys–Dietz syndrome Type IV
TGFB3 (NM_003239.4)	TGF-β3	TAAD, AAA/dissection, other arterial aneurysms Loeys–Dietz syndrome Type V
TGFBR1 (NM_004612.2)	TGF-β receptor Type I	TAAD, AAA, other arterial aneurysms Loeys–Dietz syndrome Type I
TGFBR2 (NM_003242.5)	TGF- β receptor type II	TAAD, AAA, other arterial aneurysms Loeys–Dietz syndrome type II

**Table 3:** Classification of gene mutations.

Class 1	Non-disease-causing/non-pathogenic or clinically irrelevant
Class 2	Likely non-disease-causing/non-pathogenic or clinically irrelevant
Class 3	Unclassified variant
Class 4	Likely disease-causing and pathogenic
Class 5	Disease-causing/pathogenic

**Table 4:** Demographic data, risk factors, and comorbidities of the patient cohort (n.s., not significant; TAD, thoracic aortic dissection; TAA, thoracic aortic aneurysm).

Patient Characteristics		Men	Women	*p*-Value
Number *n* (%)	65 (100)	50 (76.9)	15 (23.1)	
Age (in years)	48.4 ± 10.6	48.1 ± 10.3	49.6 ± 12.1	n.s., *p* = 0.635
TAD *n* (%)	36 (55.4) 28 (77.8) type A aortic dissection 8 (22.2) non-A, non-B aortic dissection	28 (77.8)	8 (22.2)	n.s., *p* = 0.830
TAA *n* (%)	29 (44.6)	22 (75.9)	7 (24.1)	
Performed surgical procedures in TAD In all cases, treatment of acute disease	Only ascending aortic replacement: 2 (5.6) Bentall/David technique: 16 (44.4) + Hemiarch replacement: 12 (33.3) Frozen elephant trunk: 6 (16.7)			
Performed surgical procedures in TAA Acute disease: 11 (37.9) Chronic disease: 18 (62.1)	Root remodeling: 1 (3.5) Bentall/David technique: 17 (58.6) + Hemiarch replacement: 6 (20.7) Aortic arch replacement: 5 (17.2)			
Bicuspid aortic valve	7 (10.8)			
Aortic regurgitation With bicuspid aortic valve	12 of the total 65 patients (18.5) 4 of 7 patients (57.1)			
Cardiovascular risk factors	*n* (in %)			
Arterial hypertension	52 (80.0)			
Former or current smoking status	19 (29.2)			
Hyperlipidemia	10 (15.4)			
Diabetes mellitus	4 (6.2)			
Comorbidities				
Coronary heart disease	8 (12.3)			
Carotid stenosis/stroke	3 (4.6)			
Peripheral arterial disease	1 (1.5)			
Chronic renal insufficiency	5 (7.7)			
COPD	1 (1.5)			

**Table 5:** Evaluation of the histological criteria for connective tissue diseases in patients with thoracic dissection or aneurysm.

Diagnostic Criteria		*n* (%)
All histological signs	Summary assessment by the pathologist as to whether the findings may correspond to a connective tissue disorder	29 (61.7)
Overall media degeneration	Without differentiation	38 (80.9)
Changes in elastic fibers	Total	38 (80.9)
	Elastic fiber fragmentation and/or loss (EFF)	30 (63.8)
	Elastic fiber thinning	3 (6.4)
	Elastic fiber disorganization (EFD)	5 (10.6)
Changes in the extracellular matrix	Mucoid extracellular matrix accumulation (MEMA)	31 (66.0)
Grading	Mild	8 (17.0)
	Moderate	8 (17.0)
	Severe	15 (31.9)
Presence of atherosclerosis	Total	24 (51.1)
Grading	Mild	17 (36.2)
	Moderate	3 (6.4)
	Severe	4 (8.5)
Changes in smooth muscle cells	Smooth muscle cell nucleus loss (SMCL) Laminar medial collapse (LMC) Smooth muscle cell disorganization	3 (6.4)
Changes in collagen tissue	Without differentiation	1 (2.1)

**Table 6:** Distribution of gene mutations across the study cohort; 3 of 30 patients had mutations in two different gene locations (n.s., not significant).

	*n* (%)	Men *n* (%)	Women *n* (%)	*p*-Value
Patients	65 (100)	50 (76.9)	15 (23.1)	n.s., *p* = 0.525
Detected mutation	30 (46.2)	22 (73.3)	8 (26.7)	
No mutation detected	35	28 (80.0)	7 (20.0)	
Mean age (in years)				
Total cohort	48.4 ± 10.6			n.s., *p* = 0.039
Patients with mutation	46.0 ± 11.8			
Patients without mutation	50.5 ± 9.2			

**Table 7:** Detected classic mutations and genetic variants in the study cohort (n.s., not significant).

Name of the Gene	Number of Mutation Detections *n* (%)	Classic Mutation *n* (%)	Mutation Variants *n* (%)
FBN1	10 (33.3)	2 (6.7)	8 (26.7)
MYH11	6 (20.0)		6 (20.0)
TGFB2	3 (10.0)		3 (10.0)
MYLK	2 (6.7)		2 (6.7)
NOTCH1	2 (6.7)		2 (6.7)
TGFBR1	2 (6.7)		2 (6.7)
ACTA2	1 (3.3)		1 (3.3)
COL3A1	1 (3.3)	1 (3.3)	
NOTCH3	1 (3.3)	1 (3.3)	
PRKG1	1 (3.3)	1 (3.3)	
SMAD3	1 (3.3)		1 (3.3)
SMAD6	1 (3.3)		1 (3.3)
TGFBR2	1 (3.3)		1 (3.3)
45, X	1 (3.3)	1 (3.3)	

## Data Availability

Study data are unavailable due to privacy or ethical restrictions.
